# Virtualized Microphone Array for Moving Sources Mapping

**DOI:** 10.3390/s25020362

**Published:** 2025-01-09

**Authors:** Francesca Sopranzetti, Alessia Caputo, Paolo Castellini

**Affiliations:** Department of Industrial Engineering and Mathematical Sciences, Polytechnic University of Marche, 60131 Ancona, Italy; f.sopranzetti@univpm.it (F.S.); a.caputo@pm.univpm.it (A.C.)

**Keywords:** virtualized array, beamforming, resolution, moving source

## Abstract

The acoustic analysis of a moving object, such as in pass-by or fly-over tests, is a very important and demanding issue. These types of analyses make it possible to characterize the machine in quite realistic conditions, but the typical difficulties related to source localization and characterization are usually exacerbated by the need to take into consideration and to compensate for the object movement. In this paper, a technique based on acoustic beamforming is proposed, which is applicable to all those cases where the object under investigation is moving. In the proposed technique, the object’s movement is not regarded as a problem but as a resource, enabling a virtual increase in the number of microphone acquisitions. For a stationary acoustic emission from a moving object, each time segment of the acquired signal is treated as if it is coming from a microphone (virtual) positioned differently relative to the object’s reference system. This paper describes the technique and presents examples of results obtained from both simulated and real signals. Performance analysis is conducted and discussed in detail.

## 1. Introduction

Since its introduction, beamforming has established itself as the leading technique for acoustic measurements on moving sources, above all because of the rapidity of measurement. As Michel recalled in [1], as soon as the measurement hardware was improved and enabled the use of higher sampling frequency and a greater number of microphones, microphone arrays started to be applied to measure the acoustic field produced by high-speed moving sources, and the measurement uncertainty [2] significantly decreased. Already in 1977, King and Bechert [3] demonstrated the capability of beamforming to identify the acoustic sources on trains at speeds up to 70 ms^−1^. In the papers following this, the attention was focused on different aspects, sush as finding the best array shape for those kinds of applications [4], or evaluating source powers by deconvolution methods [5].

Analogously, the power of beamforming as an instrument for the understanding of flight data was soon discovered by Howell in 1986 [6], who initiated the exploitation of this technique in fly-over measurements. In the last 25 years, this field, together with the more general field of aeroacoustics, has been where the major innovations in beamforming, in terms of array shape and design [7], deconvolution techniques [8], and quantification of absolute levels, have been made [9].

Meanwhile, in the last decade, the increasing interest in the acoustic characterization of wind turbines has started steering attention to the noise emission of rotating systems. One of the first experiments on a wind turbine rotor was performed by Sijstsma [10], who developed the ROSI technique to de-Dopplerize signals coming from noisy rotating blades. Recently, new techniques have been introduced to overcome the main drawbacks of the time-domain beamforming formulation needed for these applications; in particular, Camargo [11] proposed a frequency-domain technique to localize moving sources.

Apart from accounting for moving sources, all these applications have the analysis of high-frequency sounds in common. Thus, microphones constituting the array should be close enough to achieve the highest suppression of side lobes and obtain an optimal level of Signal-to-Noise Ratio (SNR), and there is also a need for large aperture arrays to obtain a resolution high enough to distinguish individual sources. In fact, according to the Rayleigh criterion [12] and considering the formulation introduced, among others, by Johnson [13], Mueller [14] and Christensen and Hald [15], the resolution for a phased microphone array can be expressed as

(1) $$R\left(\theta \right)=\frac{a}{cos^{3}\left(\theta \right)}\frac{z}{D}\lambda ,$$

where *a* is a parameter depending on the number and arrangement of microphones, *z* is the off-axis distance between the array and the source, θ is the angle of incidence of the sound wave on the array plane, *D* is the array aperture, and λ is the emission wavelength of the source. Thus, with the source emission frequency fixed, a better resolution in a specific angle of sight can be obtained only by increasing the aperture. However, in practical applications, the number of microphones is limited, and a large aperture leads to widely spaced microphones, with consequences to the maximum analysis frequency. In fact, always referring to [15], results with high SNR are obtained only for frequencies below the limit

(2) $$f_{max}=\frac{c}{\lambda_{min}}=\frac{c}{2d}$$

where *d* is the minimum spacing between array microphones.

As the number of microphones is usually a fixed constraint, if the array aperture is determined, the minimum microphone spacing is consistently limited; in this way, a larger array aperture is associated with a wider microphone spacing. In other words, favoring resolution hinders the maximum frequency.

Therefore, the spatial resolution induced by the size of the microphone array is one of the key factors for the goodness of beamforming results and has always been investigated. At first, solutions focused on finding the best geometry for the array [16]. In the last decade, however, new algorithms working on the correction of the spatial filtering performed by the array itself have been introduced. Among these, the most important are Brook’s and Humphrey’s DAMAS [17], Dougherty’s DAMAS2 [18], and Sijstma’s CLEAN-SC [19], all operating on the beamforming output. Fleury and Bulté extended those techniques to applications with moving sources [20].

More recently, different solutions were found for stationary cases by Castellini and Sassaroli in [21,22]. The authors first introduced the concept of average beamforming. They then suggested to perform multiple acquisitions with the same array by moving it each time to a different location within the volume under investigation. They acquired the signal of a fixed reference microphone simultaneously and used it to perform the phase realignment of the array data set to obtain a global cross-spectral matrix. In this way, the single array acquisitions were joined together as if they were acquired simultaneously, i.e., as if a unique global array resulting from the combination of the single ones was used. Being the extension of the global array larger, the overall resolution increased.

In [23], a hybrid beamforming method is presented for application to moving sources with a short pass-by window. This method, based on a combination of features of time- and frequency-domain methods, is applied to railway pass-by measurements.

Differently, Bryden in [24] and Cigada et al. in [25] suggested using a moving microphone array approach instead of the multistep one characteristic of the average beamforming. Their idea was to increase the number of measurement points by actuating, with continuous motion, the frame supporting the array during the acquisition process. Due to the array’s movement, each pressure value was sampled at different positions along the trajectory described by each microphone, making it possible to increase the number of microphones in a fictitious way. Then, the authors removed the motion effect from the array signals by applying time-domain beamforming. Since they actually performed beamforming, they needed to sample the sound field with a microphone array. Similarly, Comesana [26] adopted the so-called Scan&Paint technique, where a single PU-intensity probe is moved, scanning the area of interest, while two microphones are fixed in the far field for the phase realignment. However, the movement of the array can add difficulties to the experimental setup’s design because of the cables and the movement system. All things considered, the array should consist of hundreds of microphones, but that would lead to a several drawbacks, such as higher costs, higher performance requirements for the acquisition system, and increasing technical difficulties, for instance, in the management of all the cables.

From this perspective, the technique proposed in this paper makes it possible to originally bypass the hurdles mentioned above. In fact, it uses knowledge of the object trajectory to virtually extend the beamforming array used in the direction of the object’s motion: for instance, if a 1D array is used for a pass-by measurement, the array is virtually expanded to become a 2D array. At the limit, a single microphone can also be virtualized to a 1D array. It is evident hereinafter that the source motion is an essential factor for the proposed method to succeed. This aspect is a fundamental difference when compared to the other state-of-the-art techniques which need to remove the source motion, with this being only a necessary measurement condition for the phenomenon under investigation to occur: for example, an airplane must be in flight for the sound to be detected in operating conditions.

In [27], the idea of a mobile antenna for the evaluation and performance improvement of Direction of Arrival (DoA) is discussed. The tensor decomposition-based method for estimating the parameters of multi-path channel components is applied on a non-acoustic case (Wi-Fi transmitting and receiving).

For the state of the art, the idea of mathematically enhancing a microphone array has been introduced in direction of arrival (DOA) problems [28,29,30]. Doblinger [31] extended the idea of an interpolated array to acoustic beamforming applications. Practically, he operates on the cross-spectral matrix acquired by means of an appropriate transformation that makes it possible to obtain a desired Point Spread Function (PSF). However, this technique is applicable only in the case of static sources and requires a complete microphone array. On the contrary, the idea proposed hereinafter is to exploit the source motion and can work also for a single microphone acquisition, thus opening a new scenario in pass-by and fly-over measurements.

## 2. Theoretical Background

Consider a steady monopole source of strength Q(t) moving subsonically with velocity v. In the formulation described, the velocity of the vehicle was chosen to be rather low, both in absolute terms and in angular terms with respect to the physical array. This allowed each individual chunk to be treated as “quasi-stationary” and sound propagation to be simplified. Vehicles in subsonic motion, as in the cases of automotive pass-by or aircraft fly-overs, are among the most frequent and interesting, and also make the calculation of propagation simpler. The generalization of the proposed technique for the case of supersonic vehicles is possible and straightforward.

The monopole position is a function of time expressed by $x_{s}\left(t\right)$. The knowledge of the vehicle trajectory is of paramount importance in the proposed technique. The vehicle trajectory can be easily determined by supporting measurement techniques, such as the use of cameras, optical targets, and measurement of vehicle speed (usually but not necessarily constant). The monopole, i.e., a pressure point source, emits a sound perturbation propagating to an observer at x in the far field according to the law described by the Green’s propagation function for the wave equation $G(x_{s},x,t-\tau )$ [32]

(3) $$G\left(x_{s},x,t-\tau \right)=\frac{1}{4\pi |x_{s}-x|}\delta (t-\tau -|x_{s}-x\left|/c_{0}\right).$$

Equation (3) represents an impulsive, spherically symmetric wave expanding from the source at $x_{s}$ at the speed of sound $c_{0}$.

Thus, the pressure due to the monopole considered perceived by the observer at x can be expressed as follows:

(4) $$\begin{array}{cc} p(x,t) & =\frac{1}{4\pi }\int_{-\infty }^{\infty }\frac{Q\left(\tau \right)\delta (t-\tau -|x-x_{s}\left(\tau \right)|/c_{0})}{|x-x_{s}\left(\tau \right)|}d\tau \\ \; & =\frac{Q(\tau_{e})}{4\pi |x-x_{s}\left(\tau_{e}\right)\left|\right|\frac{\partial }{\partial \tau }(t-\tau -|x-x_{s}\left(\tau \right)|/c_{0}{)|}_{\tau =\tau_{e}}}. \end{array}$$

Here, the emission time $\tau_{e}$ can be obtained as the solution of

(5) $$c_{0}\left(t-\tau_{e}\right)=\left|x-s\left(\tau_{e}\right)\right|.$$

In other words, the sound received by the microphone at time *t* was emitted by the source at a time $\tau_{e}$, where the quantity $\Delta t=t-\tau_{e}=\frac{|x-x_{s}\left(\tau_{e}\right)|}{c_{0}}=\frac{R}{c_{0}}$ is the interval needed by the sound to propagate from the source to the microphone. If the source is stationary, then the position $x_{s}\left(t\right)$ is constant for each time *t*, i.e., $R/c_{0}=const,\;\forall t$, thus $t=\tau_{e}+const,\;\forall t$. In the case of a moving source, instead, the source-to-observer relative position changes at each emission time.

By considering a subsonic motion and recalling the definition of Mach vector in Equation (6)

(6) $$\begin{array}{c} \left\{\begin{array}{c} M=\frac{v}{c_{0}}\\ M=\left|M\right|=\frac{\left|v\right|}{c_{0}} \end{array}\right. \end{array}$$

the Equation (4) can be rewritten as

(7) $$p\left(x,t\right)=\frac{Q(t-R/c_{0})}{4\pi R(1-M\cos\theta )},$$

where θ indicates the angle between the direction of observation and the source motion one. Compare now Equation (7) to the one describing the sound field of a stationary monopole (Equation (8))

(8) $$p\left(x,t\right)=\frac{Q(t)}{4\pi R}.$$

It can be seen that the source motion affects the actual amplitude received by the microphone by a factor (1−Mcosθ), called the Doppler factor, which depends on the relative speed between the source and observer in the direction of observation. As is well known, the Doppler effect also leads to a variation in the actual frequency of the sound perceived by the observer. In particular, the Doppler frequency $f_{D}$ can be expressed as a function of the actual source frequency *f* by Equation (9)

(9) $$f_{D}=\frac{f}{\left(1-M\cos\theta \right)}$$

The problem of beamforming on moving sources is well-known in the state of the art and can be easily solved by the time-domain formulation of the delay&sum algorithm [33]. Practically, three steps are needed:

1. Sound field discretization in a series of focus points, each representing a monopole source, the power of which is estimated by the following steps. It should be noted that focus points cover the area in which the emitting object is supposed to be localized: they constitute an investigation surface, fixed to the object (i.e., moving with it), within which the real source is searched.
2. De-Dopplerization of the signals acquired by the microphones of the array. This step is necessary to remove the effect of the object’s motion from the acquired signals. In other words, this step involves changing the reference system to one fixed on the moving object to simulate the case of a static object. Practically, a focus point at a time is considered as the actual source; source-to-observer propagation delays are calculated and compensated to virtually align the array microphones in the source’s direction of sight (i.e., the focus point being considered).
3. Sum of de-Dopplerized signals, obtained by considering one focus point at a time. This step exploits the concept of constructive and destructive interference: signals that are correctly de-Dopplerized correspond to microphones correctly aligned and distributed on the same wavefront of the source’s sound. Their signals are in phase, constructively interfering to yield a high power estimate for the monopole at the focus point being considered. Conversely, if the array is focused on a focus point where there is no source, the microphone signals are out of phase and destructively interfere, resulting in a low power estimate for the monopole.

To achieve the correct de-Dopplerization of the signal, a precise knowledge of the object’s position at the time of acquisition is essential so that the exact source-to-microphone propagation delays can be assessed. One of the most critical aspects of this step is the signal re-interpolation needed to implement the correct delays. Howell [6] showed that a higher SNR can be obtained by measuring with a sampling frequency at least five times higher than the maximum frequency of interest.

The technique proposed here introduces a new way to fully exploit the de-Dopplerized signals by adding a further step before summing them together. In practice, a virtual array of sensors with an aperture as large as the source trajectory extent is obtained, thereby increasing the actual resolution of beamforming results.

## 3. Measurement Technique Description

In this section, the proposed technique is described.

**Figure 1:**
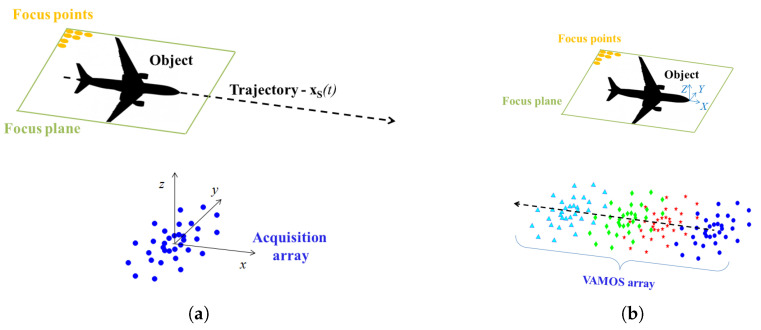
Traditional (**a**) _*vs*_ proposed approach (**b**) in fly-over test.

Consider the fly-over measurement sketched in Figure 1. The airplane is the object under investigation and is moving along the trajectory $x_{S}$. Its acoustic emission is not changing in time. A beamforming array, called the acquisition array, is measuring the sound field on the ground.

In Figure 1, it is also shown how the fly-over experiment appears if the procedure is applied. Practically, it changes the reference system to be fixed on the airplane, which thus sees the acquisition array moving with the same, but opposite, direction.

The proposed procedure consists of obtaining a virtualized array that extends along the object trajectory and is composed of a much larger number of sensors, for each of which the sound pressure signal that would be acquired if the virtual microphones were real is assessed.

Both the cases of linear and rotational motion can be treated. The linear case is discussed in detail, while the straightforward extension to the rotational case is addressed further on.

### Linear Motion

**Figure 2:**
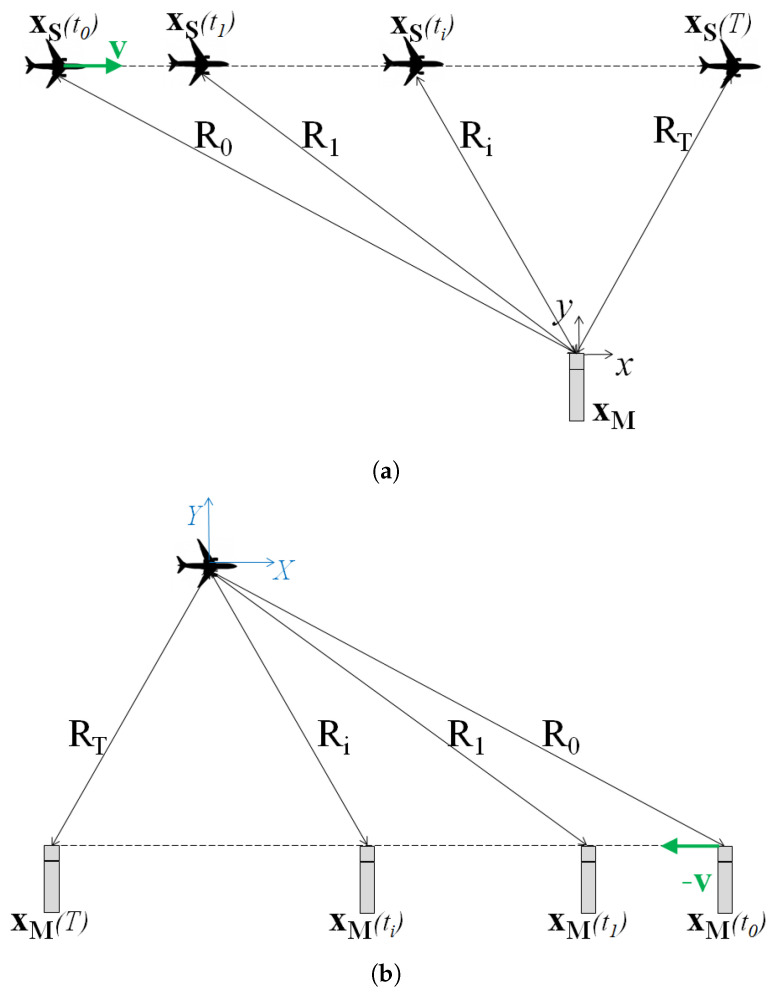
Scheme of a pass-by measurement. (**a**) Reference frame fixed on the microphone, (**b**) reference frame fixed on the source.

**Figure 3:**
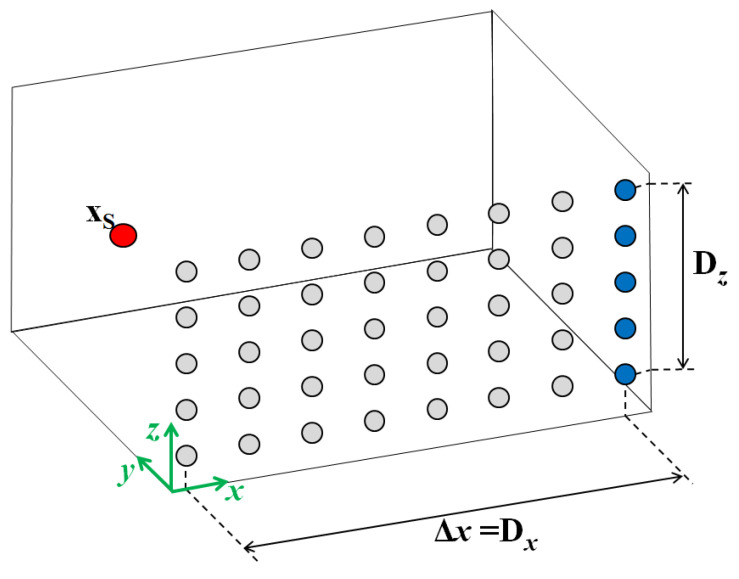
Example of a virtual array obtained by applying the proposed procedure to a ULA array extending in the z-direction. The source is considered fixed at $x_{S}=x_{S}\left(t_{0}\right)$.

With respect to the conventional implementation of beamforming on moving sources, the proposed procedure presents more complicated data processing that can be summarized, in practice, in seven steps:

1. Step 1: Fly-over (or pass-by) measurement is performed
  Consider the case in which an object, emitting a sound at frequency *f*, is moving with a velocity v and whose related sound field is sampled by a microphone array. The array is virtualized in the direction of −v and therefore, at the limit, a complete array can be obtained by the acquisition of a single microphone. Hence, without a loss of generality, the simplest case is considered in the following, as the extension to an array composed of more real microphones is straightforward.
  In the example studied, the object velocity has only one component different from zero, i.e., v={v;0;0}, and the sound field is sampled by a single microphone at $x_{M}$ with a sampling frequency $f_{s}$ for an observation time *T*.
  The simultaneous acquisition of both the sound pressure and the actual airplane position is performed so that the object position is known at all times and is synchronized with the sound pressure acquisition.
  Let $Q(\tau_{e})$ be the strength of the monopole source at position $x_{S}$ at the emission time $\tau_{e}$. The signal $p_{M}$ measured by the microphone at $x_{M}$ is expressed by Equation (7). In other words, that equation can be written as the following formulation [33]:
  (10) $$\begin{array}{c} \left\{\begin{array}{c} p_{M}\left(x_{M},t\right)=\frac{Q(\tau_{e})}{F(x_{s},x_{M},\tau_{e},t)}\\ F\left(x_{s},x_{M},\tau_{e},t\right)=\frac{Q}{p_{M}}=4\pi R\left(1-M\cos\theta \right) \end{array}\right. \end{array}$$
  where *F* is the moving monopole-to-microphone transfer function, which also evaluates the Doppler amplification. The Doppler frequency shift, on the other hand, can be corrected by properly considering the times in *Q* and $p_{M}$; by considering the monopole radiation at equally spaced emission times $\tau_{e}$, the microphone receives the signal at the time *t*, which is equal to the time needed for the sound field to propagate from the source to the microphone at the considered instant $\tau_{e}$.
2. Step 2: Time history separation into segments
  The sound pressure signal and actual object position are separated into subsequent time segments. In each segment, the airplane flies from a point, namely $x_{S0}$ or $x_{S1}$, to another at a distance depending on both v and $T_{k}$.
  Theoretically, there are no constraints for the time segment selection: they can be of different lengths, with or without overlapping, covering the entire observation time or not. Referring still to the simplest case, here the time segments are considered to have a constant length $T_{K}$, without overlapping, and covering the entire observation time. The number of time segments into which the time history is divided is then
  (11) $$N=\lfloor \frac{T}{T_{K}}\rfloor .$$
3. Step 3: Backward propagation
  The microphone signal is de-Dopplerized by considering the position and velocity of the object acquired. In this way, the source strength in each focus point is reconstructed by projecting it back to the object, which begins to move with it.
  The de-Dopplerized signal $p_{k,ded}$ at the real microphone is obtained by the following equation, where $R(t_{k})$ is the distance between the source and the microphone at the considered time:
  (12) $$\begin{array}{cc} p_{k,ded}\left(t_{k}\right) & =p_{M}\left(t_{k}+\Delta t_{prop,k}\right)\cdot \left(1-M\cos\theta_{k}\right)=\\ \; & =p_{M}\left(t_{k}+\frac{R(t_{k})}{c_{0}}\right)\cdot \left(1-M\cos\theta_{k}\right) \end{array}$$
  The source strength $Q_{k}=Q\left(t_{k}\right)$ is estimated by advancing the de-Dopplerized signal for the propagation time in stationary conditions, hereinafter referred to as the back-propagation time $t_{bp}$
  (13) $$\begin{array}{c} \left\{\begin{array}{c} Q_{k}\left(\tau_{e,k}\right)=p_{k,ded}\left(t_{k}-t_{bp}\right)\\ t_{bp}=\frac{R(k\cdot T_{k})}{c_{0}} \end{array}\right. \end{array}$$
  where the source strength is expressed in terms of the *k*th emission times $\tau_{e,k}$. After the de-Dopplerization, the situation is re-created where the source is fixed at $x_{S0}$ and the microphone is fixed at $x_{M}$.
  For each time segment *k*, the signal acquired by the microphone and the source trajectory are, respectively:
  (14) $$\begin{array}{c} \left\{\begin{array}{cc} p_{k}=p_{M}\left(t_{k}\right) & \\ x_{k}=x_{s}\left(t_{k}\right) & \end{array}\right.for\;t_{k}=\tau_{e}=k\cdot T_{K},\ldots \left(k+1\right)\cdot T_{k}-1. \end{array}$$
  In Equation (14), it is assumed that the acquisition time *t* coincides with the emission time $\tau_{e}$.
4. Step 4: Virtualized array definition
  In practice, by considering each time segment separately, it is as if the entire experiment is actually split in a number of sub-experiments, each extending for an observation time coincident with $T_{k}$, with each subsequent and not simultaneous, and with each referred to different source positions, and consequently, to different source-to-microphone positions. This condition makes it possible to consider, in the moving object reference system, each time a different segment acquired in a different position, in which we can imagine a virtual array is placed Figure 2. Subsequently, the number of virtual microphones obtained is equal to N. If the monopole is moving with linear motion, the arrangement of the microphones in this case is a uniform linear array (ULA) in the direction of the motion. The spacing between two adjacent microphones is equal to $\Delta x=\left||v|\right|\cdot T_{K}$.
  A virtual microphone is associated with each time segment. To locate it, it is necessary to consider the sub-experiment that the time segment is referring to. In practice, the initial object-to-microphone vector distance of the sub-experiment is considered. The corresponding virtual microphone location, $x_{\mathrm{Mi}}$, is obtained by fixing $-R_{i}$ on the reference object position $x_{S0}$.
5. Step 5: Forward propagation
  Virtual microphones can be placed at $x_{M0}$, $x_{M1}$, $x_{M2}$, and so on, and the sound pressure signal they would have acquired can be obtained from the source strength at each time segment by using the Green’s propagation function, considering the source stationary in $x_{S0}$.
  The signal on the virtual microphone $p_{Mvk}$ is then calculated by applying the Green’s propagation function to the source strength:
  (15) $$p_{Mvk}\left(t_{k}\right)=\frac{Q_{k}\left(\tau_{e,k}+\frac{R_{k}}{c_{0}}\right)}{4\pi R_{k}}$$
  where $R_{k}$ is the distance between the source and the virtual microphone. By repeating this procedure for all the N time segments, N virtualized microphones can be obtained with their relative time histories.
6. Step 6: Synchronization of virtual microphones
  The different sub-experiments are asynchronous, but subsequent, and, above all, derive from the same acquisition: therefore, the phase relations among them are known. This makes it possible to re-synchronize the virtual signals so that the time vector becomes the same for all of them, and they are corrected in terms of relative phase. To reproduce the simultaneity of the virtual microphone acquisition, the delays among the time segments also need to be compensated. This can be completed by simply advancing the signals obtained so far by the proper quantity, as shown in the following equation:
  (16) $$\begin{array}{c} \begin{array}{cc} p_{Mvk}\left(\tau_{e}\right)=p_{Mvk}\left(t_{k}-k\cdot T_{K}\right) & \mathrm{for}\;k=1,\cdots K. \end{array} \end{array}$$
  It can be noticed that the time segments originate from a single acquisition; thus, the synchronization can be performed using the equation above, without the need for a sync signal, such as a trigger.
  Equation (16) collects the signals of the virtual array and represents the sound pressures that would be acquired by the array if it were real and the source were fixed at $x_{S}$. To localize the sources, beamforming should be performed by considering the source at each time in a different focus point. In other words, this procedure should be repeated for each focus point; thus, for each focus point, a set of $p_{Mvk}$ signals is obtained. The method to combine these signals is detailed below.
  In the more general case of an acquisition array composed of $N_{acq}$ microphones, the virtual array is composed of $N\cdot N_{acq}$ sensors. For instance, consider Figure 3, where the microphone array used for the measurement is a line along the y-direction, and the source is moving in the x-direction. During *T*, the source covers the space Δx. By applying the proposed procedure with uniform time segment length and without overlap, the virtual microphones added to the array are the gray ones. The overall array gains a new large aperture along the x-axis, increasing the resolution in that direction.
7. Step 7: Beamforming and sources localization in the proposed procedure
  From the previous steps, the virtual signals are obtained. The number of microphones constituting the array is equal to the number of time segments considered in the observation time. The virtual signals can be exploited to perform beamforming and localize acoustic sources within the focus plane over the object.
  Actually, all the beamforming techniques, including deconvolution methods, can be applied to the proposed procedure’s output. However, they all need a re-formulation, because a set of virtual signals is obtained for each focus point. In the following, the conventional beamforming (CB) technique is formulated, as it is the most frequently used technique.
  The starting point is the same as for all the beamforming techniques: the sound field, where the source is assumed to be, is discretized into a series of *L* focus points, each representing an emitting monopole and moving like the object. Each focus point is considered as the actual source in the proposed procedure. In other words, the procedure from step 2 to step 4 should be repeated once for each focus point. Each time, the source considered coinciding with one of the focus points, so the de-Dopplerization, backward propagation, and forward propagation should be performed assuming that the actual source is the focus point considered.
  According to the previously mentioned procedure, the virtualized array is formally obtained only in step 4th. However, once the time segment length has been decided, the set of virtual microphones can be derived only once from the information about the object’s trajectory. In fact, the input necessary to obtain the microphone arrangement is just the variation in the object–microphone distance, which is constant for all focus points, as the focus plane is built with the object itself.
  For each focus point, the signals $p_{Mvk}\left(\tau_{e}\right)$ from Equation (16) are estimated and indicated as $p_{l}$, where the subscript *l* indicates that this set of virtual signals was obtained for the *l*th focus point. Similarly, from the virtual signals, the *l*th cross-spectral matrix can be obtained as
  (17) $$\begin{array}{c} {\mathrm{CSM}}_{l}=\frac{1}{2}p_{l}p_{l}^{*} \end{array}$$
  where $p_{l}$ is the spectral representation of $p_{l}$.
  By defining the steering vector to the *l*th focus point as
  (18) $$\begin{array}{c} g_{l}={\left\{\frac{e^{-i\frac{2\pi fR_{k}}{c_{0}}}}{4\pi R_{k}}\right\}}_{k=1,\cdots N} \end{array}$$
  the amplitude of the monopole at the *l*th focus point can be obtained using the CB equations [33]:
  (19) $$\begin{array}{c} A_{l}=\frac{g_{l}^{*}\;{\mathrm{CSM}}_{l}\;g_{l}}{∥g_{l}{∥}^{4}}. \end{array}$$
  As is commonly performed in CB, by removing the autospectra from the ${\mathrm{CSM}}_{l}$, the SNR of the maps increases, as the self-noise on the microphones is removed. The map obtained is similar to the one from CB, and a deconvolution algorithm can be applied to filter the array PSF and increase the final SNR.

#### Rotational Motion

**Figure 4:**
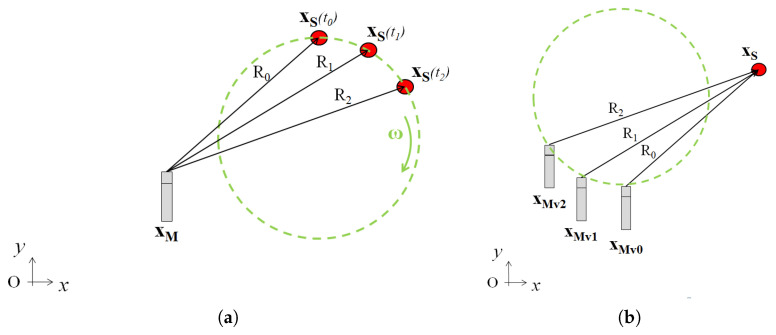
Scheme of the proposed procedure approach on a rotating object (**a**) Source motion, (**b**) Virtual array.

The extension to the case of the source in rotational motion is straightforward. With reference to Figure 4, the source at $x_{s}$ is moving with an angular velocity ω, and a microphone is fixed at $x_{M}$. The relative position between the source and the microphone changes, and this can be exploited to virtualize the real microphone acquisition. Even in this case, the acquisition time is split into N time segments, and the N virtual microphones are obtained as shown in the figure.

From an analytical point of view, the formulation remains the same as expressed in Equations (14)–(16), with the only difference being that the Mach number vectors have two components that change each time.

## 4. Results and Discussion

### 4.1. Numerical Simulation

**Figure 5:**
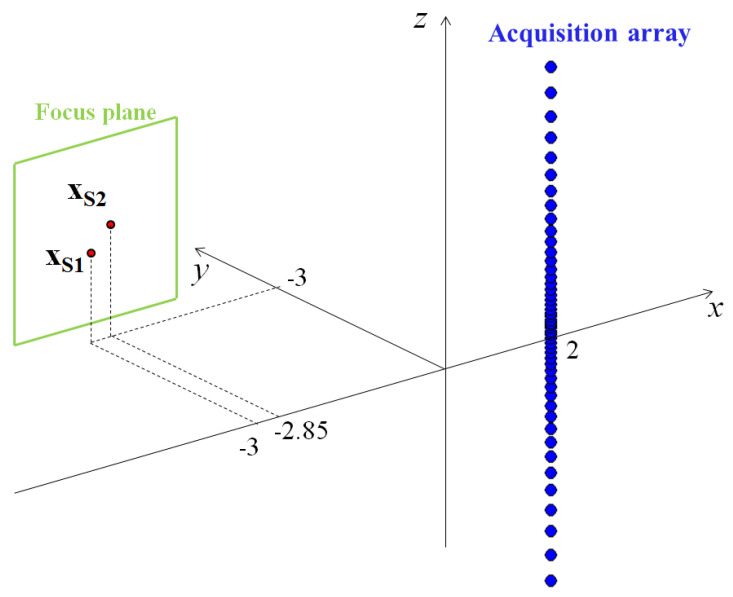
Illustration of simulated conditions.

In order to demonstrate the performances of the proposed procedure and to analyze the limits and sensitivity of the technique, a numerical model has been developed. The simulated conditions are illustrated in Figure 5: two monopole sources were considered, moving with the same velocity $v=10\;{\mathrm{ms}}^{-1}$ and emitting at the same frequency f=2 kHz. Their distance in the x direction is equal to 0.15 m, and in the y direction, it is 0.3 m. All the beamforming maps of beamforming were calculated on a focus plane with a resolution of 0.02 m. Up to this point, the analysis was performed by considering a single microphone acquisition, which, when virtualized, gives rise to a uniformly spaced linear array. In this subsection, the investigation progresses to actually check the resolution of the virtual array. Unlike before, a multiple sensor acquisition array is considered.

#### 4.1.1. Resolution Capability and Comparison with Standard Techniques

In order to verify the actual performances obtainable through the proposed procedure, these results were compared with those achievable using state-of-the-art techniques. Since the procedure can be applied to pass-by or fly-over measurements, the state-of-the-art technique chosen for comparison is the classical time-domain beamforming with different array configurations.

In this paper, we chose to compare results of beamforming in its classical and simplest formulation (Delay-and-Sum) to highlight potential limitations of the proposed technique, which would be hidden by more sophisticated beamforming formulations.

**Figure 6:**
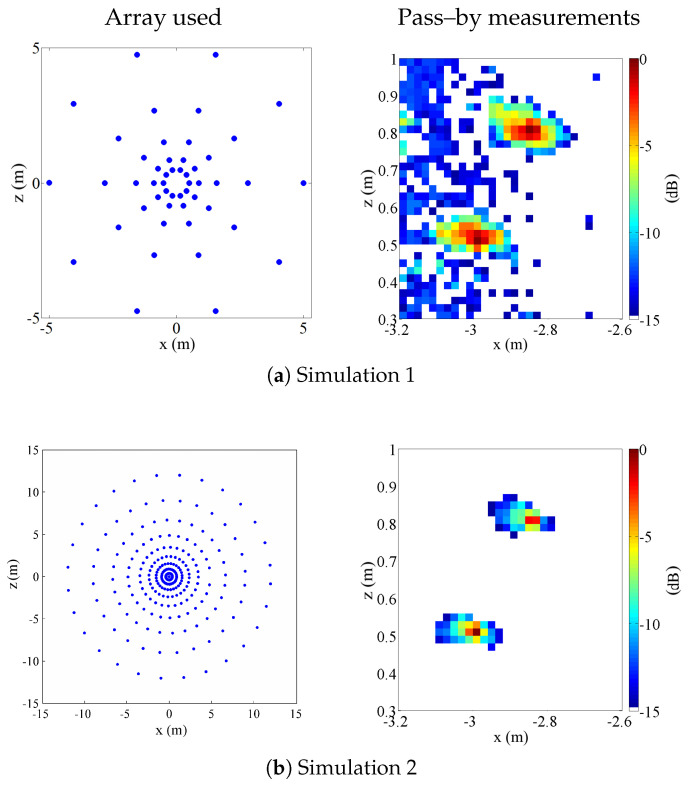

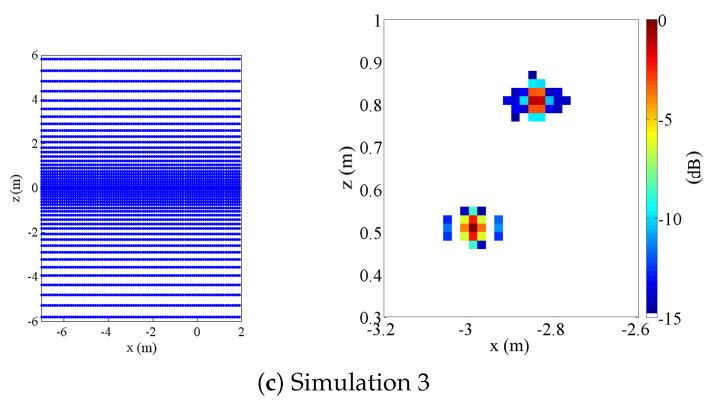
Results obtained for simulations 1 (**a**), 2 (**b**), and 3 (**c**) in pass-by.

The proposed procedure makes it possible to increase the acoustic information collected from the object: data can then be processed using typical algorithms used in beamforming. The conditions that the array must satisfy are dependent on the post-processing strategies and not the virtualization process. While the position of the microphones in the direction transverse to motion is dictated only by their physical location, the position in the direction of motion certainly depends on the speed of the vehicle but can also be changed at will through a different choice of the instants at which each chunk of the signal, which determines a new set of virtual microphones, is chosen. This makes it possible, from the same data acquired in the test, to construct a different array depending on the beamforming algorithm chosen. Regarding this, it is also noted that inverse beamforming techniques have much less demanding microphone distribution requirements, simplifying array construction. For the same reasons, the proposed technique is compatible with any type of sound (multi-tonal, harmonic or noise sources) because the first part of the processing operates in the time domain, and the difference between different sounds could impact the later processing, regardless of the virtual microphones procedure. In particular, three situations were considered:

1. Standard array with $N_{acq}=$ 50: the acquisition array is composed of 50 microphones, i.e., the same number of microphones as the acquisition array used in case 3. They are organized in a typical arrangement for pass-by measurements, as shown in Figure 6. The array covers an aperture of 10 m, which is a compromise, for the given small number of microphones, between resolution and SNR at high frequencies.
2. State-of-the-art array simulating the same array used by Sijtsma in [9] in the literature with $N_{acq}=243$: the acquisition array is organized in an arrangement suited for the pass-by application (see Figure 6). It is a spiral array composed of 243 microphones within a radius of 12 m.
3. The proposed procedure test with $N_{acq}=50$: the experiment is depicted in Figure 6. The acquisition array simulated is composed of 50 microphones arranged logarithmically along the z-direction, covering a 12 m aperture. $N_{acq}=50$ was chosen in order to obtain a good SNR, but this number can be further optimized. The array is positioned symmetrically with respect to the x-axis. The proposed procedure was applied by considering $T_{k}=0.01$ s, without overlapping, and by considering Equation (11) as valid. The virtualized array obtained is thus composed of 4450 microphones arranged logarithmically along the y-direction and uniformly along the x-direction. The aperture gained in the x-direction is about 9 m, while the aperture in the z-direction remains unchanged.

Figure 6 also presents the comparison among the three different situations: the column on the left illustrates the three different arrays used, while the one on the right shows the results of the pass-by measurements.

It can be clearly seen that all the techniques compared correctly localized both sources and that the source main lobes are quite sharp. The advantages of the proposed technique are also evident. If we compare the proposed procedure to the standard array, the improvement in the SNR is clear. The virtual microphones have a strong impact on enhancing the acquisition, and the noise floor is much lower. In addition, the resolution is quite different, because in the standard array, it is necessary to distribute the available microphones in a 2D pattern, so the overall size of the array must be relatively small to guarantee an adequately small average distance between each microphone. On the other hand, if we compare the proposed procedure results to that of the state-of-art array, it is evident that the quality of the maps is quite similar, both in terms of SNR and resolution, despite a significant difference in the number of microphone which is five times smaller in the proposed procedure.

**Figure 7:**
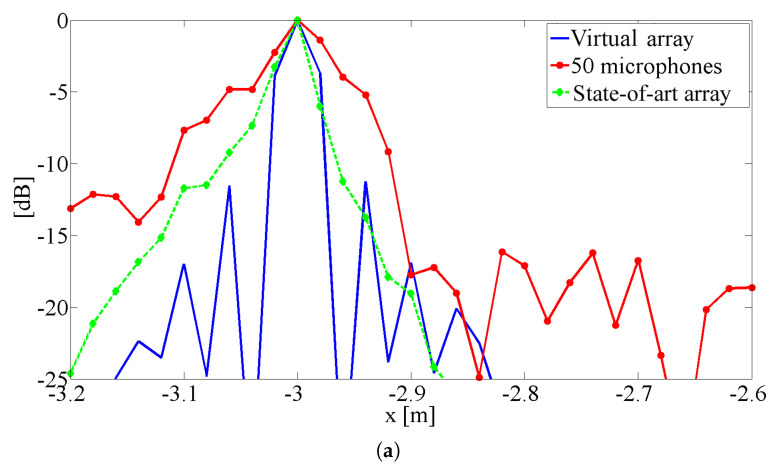

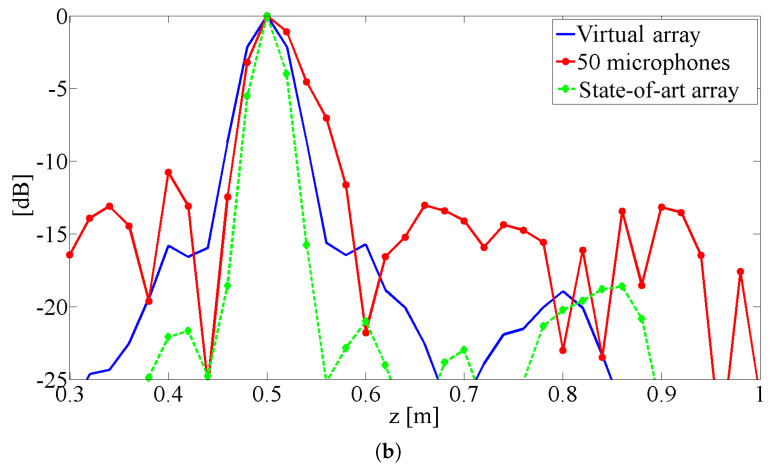
Sections of Figure 6 results: (**a**) along the x direction, (**b**) along the z direction.

In Figure 7, the same results are shown as sections passing through one source (the behaviour across the other is quite similar). Again, it is evident that the proposed procedure’s results are quite similar to those of the state-of-the-art array and significantly better than those of the standard array. On the other hand, the section in x direction shows evident sidelobes in the plot. In this implementation, the proposed procedure generates a uniformly spaced array in the direction of motion, and the sidelobes are the typical drawback. The spacing of the virtual microphone is discussed in the following section. To better understand the behavior of the proposed algorithm, the standard array, the state-of-the-art array, and the proposed procedure test were compared with steady sources, thereby eliminating contributions due to de-Dopplerization and source motion and highlight only the effect of the array shape. In a strict sense, this is a paradox for the proposed procedure, as it cannot work without source motion. To make this comparison, an array with $N_{acq}=4450$ real microphones, arranged in the same pattern as the vitual array generated by the proposed procedure, was considered. In practice, we simulated the availability of a large number of real microphones and arranged them in the same pattern as the virtual ones calculated by the proposed procedure.

**Figure 8:**
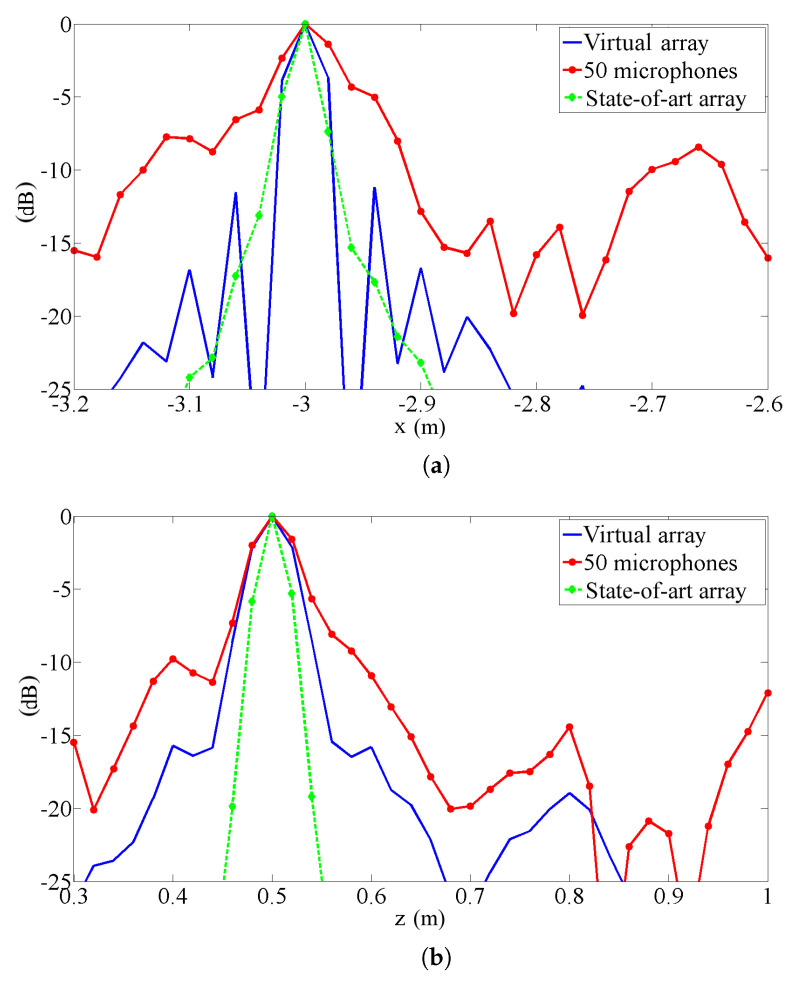
Sections of results obtained on steady source with conventional beamforming (for the proposed procedure with $N_{acq}=$ 4450 real microphones in the pattern of the array): (**a**) along the x direction, (**b**) along the z direction.

In Figure 8, the results of this simulation are shown as sections along the x and y directions. These plots confirm the results shown in Figure 7. In fact, the proposed procedure’s plot is nearly identical to the reference one, apart from a slight wake of sources in the direction of motion in the pass-by case, due to imperfect de-Dopplerization.

Both in x and in y directions, the plots are quite similar to the pass-by ones: the proposed procedure correctly calculates the positions of the virtual microphones and the related signals, so that the virtual array behaves like real ones with the same pattern.

#### 4.1.2. Spacing of Virtual Microphones: Linear Motion

Referring again to Figure 6, it can be observed that the virtual array obtained in that case has a regular pattern. This results from the selection of time segments in Equation (11). According to [14,15], the regularity of the sensor arrangement could affect the quality of beamforming results, as side-lobes may become significant.

However, this problem can be mitigated by selecting the time segments differently, such that the virtual array is not regularly spaced. For instance, one method involves fixing the coordinates of the virtual microphones along the source trajectory and then selecting the time segment accordingly. Considering the simplest case, let us refer to a 1D virtual microphone array. As an example, a logarithmic arrangement was used for the virtual array pattern along the motion direction.

In this case, the desired virtual microphone positions $\left\{x_{\mathrm{Mvk}k=1},\cdots K\right\}$ are selected within the total space traveled by the source during the acquisition time:

(20) $$\begin{array}{c} \left\{\begin{array}{c} x_{\mathrm{Mvk}}=x_{S}\left(t_{kstart}\right)\\ t_{k}\in \left[t_{kstart},t_{kstart}+T_{k}\right] \end{array}\right. \end{array}$$

From the equation above, it is clear that the length of the virtual time histories must be equal for each simulated microphone. As a result, the samples in the acquired time history are exploited in different ways:

1. The spacing between the microphones at the extremities can be larger than the distance traveled by the source during $T_{k}$, so some samples are not considered at all.
2. The spacing between central microphones can be smaller than the distance traveled by the source during $T_{k}$, so some samples are used for more than one virtual microphone, causing overlapping in the time histories.

**Figure 9:**
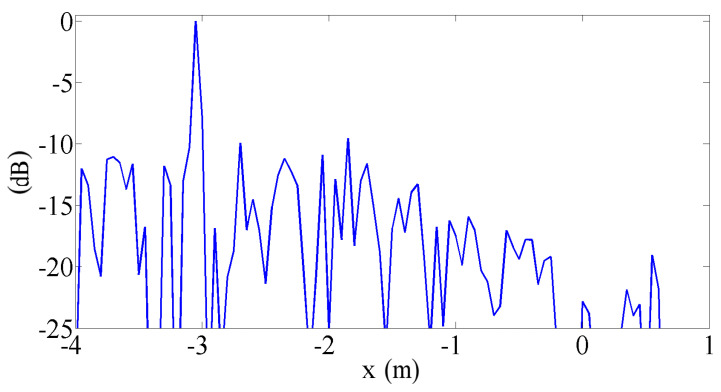
CB results: virtual array by 40 logarithmically spaced microphones.

For example, consider the simulation discussed earlier. A logarithmic array of 40 microphones was created with a time segment length of 0.01 s. The results obtained are shown in Figure 9. It can be seen that the SNR is quite low compared to typical values for a similar “real” array. This behavior is due to the central microphones being associated with time histories that overlap by 70%. This does not occur for the microphones at the extremities. Consequently, the overlapping percentage is not constant, and its low-pass filter effect impacts the microphones differently, reducing the consistency.

#### 4.1.3. Spacing of Virtual Microphones: Rotational Motion

In applications involving rotational motion, the relative position between microphones and the object is periodic if, as in common cases, the acquisition time is long enough to cover multiple rotations. In this scenario, the virtual microphones are distributed along a single circle of fixed radius. To avoid placing multiple microphones at the same position, the time segment length must be chosen appropriately. A simple but effective rule is that $\frac{\omega^{-1}}{T_{k}}$ must not be an integer. For instance, consider a source moving with an angular velocity of 50 Hz. The revolution period is $T_{p}=0.02$ s. Further consider an observation time covering two revolutions. By choosing a time segment length of 0.01 s, four microphones would be virtually obtained: the first at position $x_{\mathrm{Mv}0}$, the second at $x_{\mathrm{Mv}1}$, but then the third would coincide with $x_{\mathrm{Mv}0}$, and the fourth would coincide with $x_{\mathrm{Mv}1}$. The rule mentioned above prevents this situation.

### 4.2. Experimental Test

**Figure 10:**
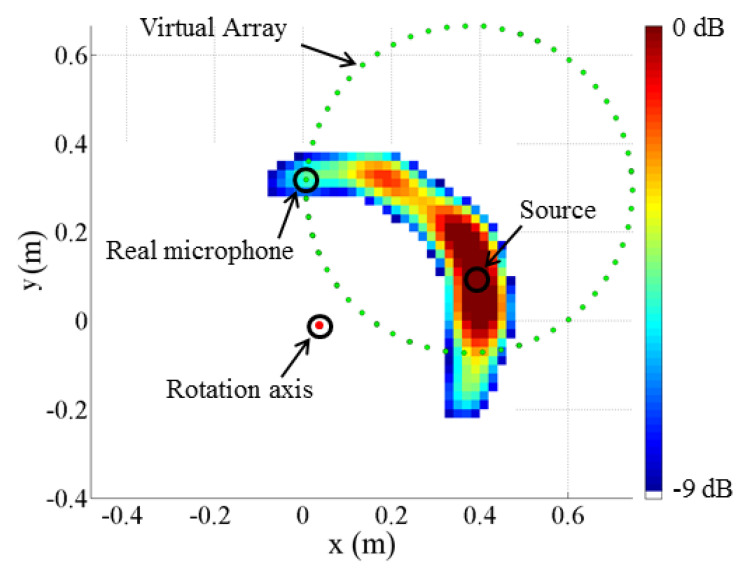
Experimental validation: the obtained virtual array and acoustic map.

To demonstrate the performance of the proposed procedure under real-world conditions, a simple test was conducted. A single-frequency sound source emitting at f=4.58 kHz was installed on a bladed disk with a radius of r=0.37 m. The disk was driven by an electric motor, with its velocity measured using an encoder with 4096 pulses per revolution. A microphone is placed 1.2 m away in the axial direction. The motor velocity was tuned such that the tangential velocity of the sound source was $v=10\;{\mathrm{ms}}^{-1}$. All beamforming maps were calculated on a focus plane with a resolution of 0.02 m. The test was performed using a single microphone acquisition, which, when virtualized, resulted in an equally spaced circular array with $N_{acq}=55$. The beamforming map obtained is shown in Figure 10. Despite using only a single microphone, the technique successfully localized the sound source. The map is well-defined, and the source is correctly identified. Additionally, the map exhibits a wake effect along the real source trajectory, highlighted by plotting the results with a dynamic of 9 dB. This effect arises due to inaccuracies in defining the relative position between the rotor and the microphone, an aspect that requires careful evaluation.

## 5. Conclusions

In this paper, a pre-processing method is presented to be applied to all those measurements in which the object under investigation is moving and emitting in a stationary way. The proposed procedure should be considered as an intermediate step between the measurement phase and the standard beamforming calculation. In fact, no differences need to be made to the standard beamforming acquisition procedure, apart from the fact that different parameters should be considered in array design, as previously mentioned. Similarly, the last step is always a beamforming calculation on the focus plane: it can be performed using any desired algorithm, with minimal effort in correctly re-writing it. It is noticed that, for common pass-by or fly-over applications, the acquisition time *T* is very long (several minutes). Such a long time history would lead to a very high frequency resolution for the subsequent beamforming analysis.

However, for practical acoustic applications, a smaller frequency resolution is preferable. This means that, even if averages are performed, only a small part of the de-Dopplerized signal is actually exploited for the beamforming analysis. By using the proposed procedure instead, the virtual time histories extend for a virtual observation time equal to $T_{k}$, significantly lower than the global observation time *T*. The proposed procedure thus makes it possible to better exploit the acquired time histories, as no information is wasted in the beamforming analysis.

The proposed procedure is compatible with different kinds of beamforming algorithms, which allow for a further resolution increase [34]. The aim of the technique is to virtually increase the array aperture by exploiting the object motion, as in case [35], to build virtual microphones. The resolution of beamforming is thus highly increased. The main difference from other techniques known in the state-of-the-art is that signals for each virtual microphone are actually obtained, which also increases the SNR of beamforming results. In this way, even with measurements performed by an array composed of a few microphones (or even a single microphone), results with high resolution and SNR can be obtained. From this perspective, the rules for the array design should change: since the proposed procedure creates the desired resolution in the direction of motion, the available microphones should be arranged along the other directions.

## Data Availability

Research data are available under request.
